# A novel small molecule agent displays potent anti-myeloma activity by inhibiting the JAK2-STAT3 signaling pathway

**DOI:** 10.18632/oncotarget.6974

**Published:** 2016-01-22

**Authors:** Zubin Zhang, Hongwu Mao, Xiaolin Du, Jingyu Zhu, Yujia Xu, Siyu Wang, Xin Xu, Peng Ji, Yang Yu, Biyin Cao, Kunkun Han, Tingjun Hou, Zhuan Xu, Yan Kong, Gaofeng Jiang, Xiaowen Tang, Chunhua Qiao, Xinliang Mao

**Affiliations:** ^1^ Jiangsu Key Laboratory of Translational Research and Therapy for Neuro-Psycho-Diseases, Department of Pharmacology, College of Pharmaceutical Sciences, Soochow University, Suzhou, China; ^2^ College of Pharmaceutical Sciences, Zhejiang University, Zhejiang, China; ^3^ Department of Hematology, The First Affiliated Hospital of Soochow University, Suzhou, China; ^4^ Department of Medicinal Chemistry, College of Pharmaceutical Sciences, Soochow University, Suzhou, China; ^5^ Department of Neurology, The First Affiliated Hospital of Soochow University, Suzhou, China; ^6^ School of Public Health, Medical College, Wuhan University of Science and Technology, Wuhan, China; ^7^ Jiangsu Key Laboratory of Preventive and Translational Medicine for Geriatric Diseases, Soochow University, Suzhou, China

**Keywords:** SC99, JAK2, STAT3, cyclin D2, multiple myeloma

## Abstract

The oncogenic STAT3 signaling pathway is emerging as a promising target for the treatment of multiple myeloma (MM). In the present study, we identified a novel STAT3 inhibitor SC99 in a target-based high throughput screen. SC99 inhibited JAK2-STAT3 activation but had no effects on other transcription factors such as NF-κB, and kinases such as AKT, ERK, and c-Src that are in association with STAT3 signaling pathway. Furthermore, SC99 downregulated the expression of STAT3-modulated genes, including *Bcl-2*, *Bcl-xL*, *VEGF*, *cyclin D2*, and *E2F-1*. By inhibiting the STAT3 signaling, SC99 induced MM cell apoptosis which could be partly abolished by the ectopic expression of STAT3. Furthermore, SC99 displayed potent anti-MM activity in two independent MM xenograft models in nude mice. Oral administration of SC99 led to marked decrease of tumor growth within 10 days at a daily dosage of 30 mg/kg, but did not raise toxic effects. Taken together, this study identified a novel oral JAK2/STAT3 inhibitor that could be developed as an anti-myeloma agent.

## INTRODUCTION

Signal transducer and activator of transcription 3 (STAT3) is an oncogenic transcription factor widely expressed in many tissues and plays an important role in regulating cellular activities. Being activated by a panel of stimuli, including cytokines, such as interleukin-6 (IL-6) [1, 2], and growth factors, such as insulin-like growth factor 1 (IGF-1) [1, 2], STAT3 forms homo- or hetero-dimers before being translocated to the nuclei where it binds to DNA and regulates gene transcription [1, 3]. STAT3 has a broad spectrum of substrate genes including anti-apoptotic *Mcl-1*, *Bcl-2*, *survivin* [4–6], and cell cycle regulators (such as *D-cyclin*s, *E2F-1*) [7, 8]. In addition, STAT3 also regulates angiogenesis via the transcription of vascular endothelial growth factor (VEGF) [9].

STAT3 is overexpressed in a broad range of cancers including hematological malignancies, including leukemia [10], lymphoma [11] and multiple myeloma (MM) [12, 13]. MM is an incurable malignancy of plasma cells. In a survey of constitutive and activated STAT3 expression and MM therapy, STAT3 was found highly expressed in more than 63% of CD138^+^ bone marrow cells of primary MM patients [14] and the highly activated STAT3 is associated with chemoresistance to most of the standard therapies [13], including dexamethasone [14]. Currently, The STAT3 signaling pathway has been proposed as a promising target for MM therapy [15]. A recent clinical trial with a STAT3 inhibitor OPB-51602 has been reported for the treatment of MM and acute myeloid leukemia [16].

D-type cyclins including cyclin D1, D2 and D3 are key members in regulating cell cycle progress. Cyclin D2 (CCND2) was reported in more than 50% of MM cell lines and primary patient cells [17] and is associated with poor prognosis [18]. We previously demonstrated that knock-down CCND2 can induce MM cell apoptosis [19]. Because CCND2 could be regulated by STAT3 [20] and this duo is critical for MM pathogenesis and poor clinical outcomes, targeting STAT3/CCND2 is an anti-MM strategy. In the present study, we designed a screen of STAT3/CCND2 inhibitors, through which a novel small molecule compound called SC99 was found to inhibit STAT3 via JAK2, a kinase that activates STAT3, and displayed oral potency in the treatment of MM.

## RESULTS

### Identification of SC99 as a STAT3 inhibitor

**Figure 1:**
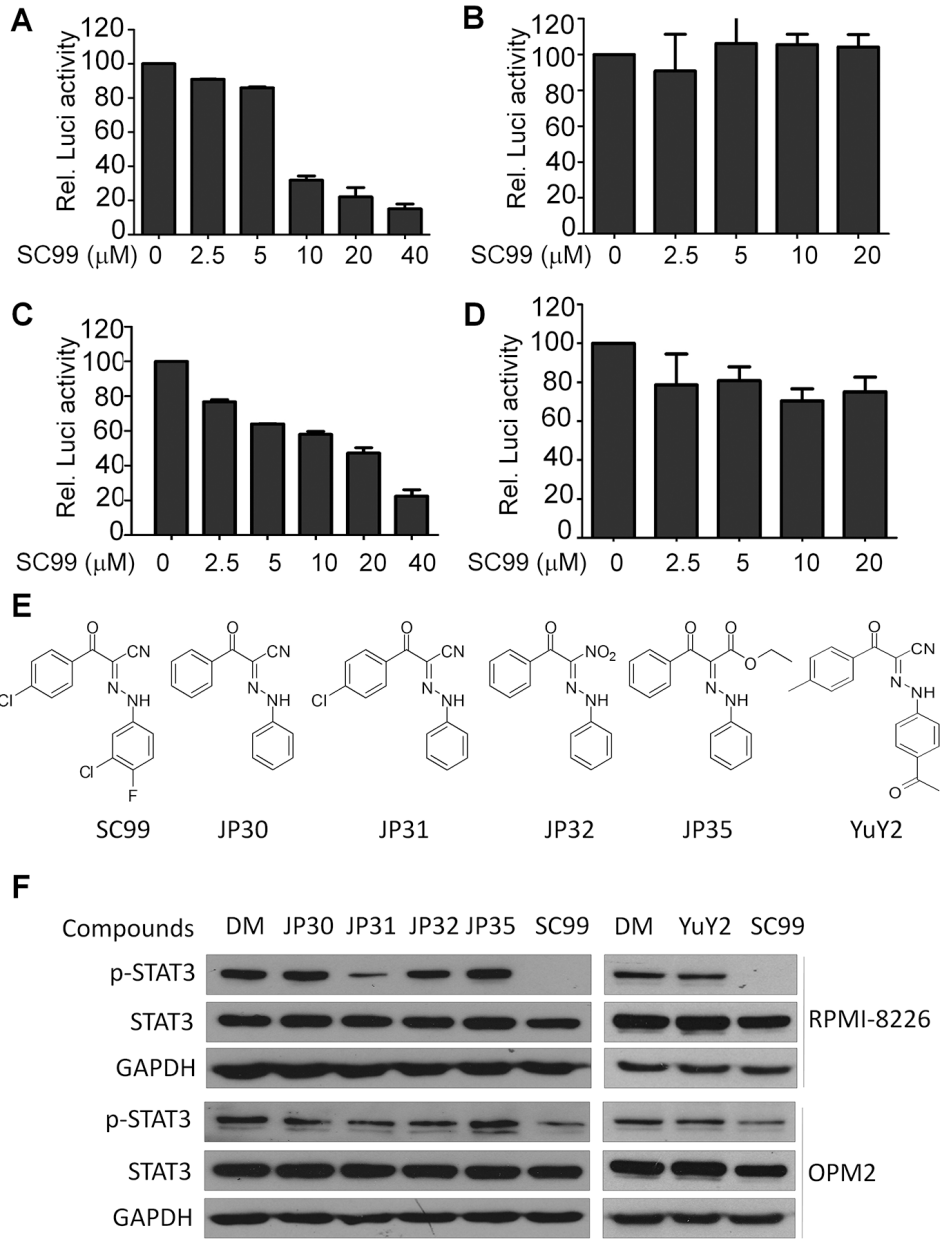
Identification of SC99 as a STAT3 inhibitor The luciferase reporter systems were established driven by STAT3 **A.** or NF-κB **B.** response elements, cyclin D2 promoter containing **C.** or lacking **D.** STAT3 response element. NIH3T3 cells expressing individual luciferase reporters were treated with SC99 for 24 hrs. Luciferase activity was evaluated with a specific substrate according to the manufacturer's instruction. **E.** a series of SC99 analogs was synthesized. **F.** MM cell lines OPM2 and RPMI-8226 were treated with each compound at 10 μM for 24 hrs, followed by immunoblotting assay against p-STAT3, STAT3 and GAPDH. DM, dimethyl sulfoxide.

To find out small molecule compounds that inhibit STAT3 activation and CCND2 transactivation, a luciferase reporter system driven by the CCND2 promoter containing a STAT3 response element was stably established in NIH3T3 cells and was applied to screen a Maybridge chemical library composed of 56000 compounds with unknown functions as described previously [21], from which SC99 was identified. SC99 inhibited STAT3-luciferase (Figure 1A) but did not inhibit luciferase activity modulated by another nuclear transcription factor NF-κB (Figure 1B). Consistent with this finding, SC99 inhibited the transactivation of CCND2 promoter which containing a STAT3 response element (Figure 1C) but failed to suppress the transactivation of CCND2 promoter which lacks a STAT3 response element (Figure 1D). These results thus suggested that SC99 was probably a STAT3 inhibitor. To optimize the structure of SC99, a panel of SC99 analogs were designed and synthesized (Figure 1E) and were applied to test their inhibitory effects on STAT3 activation at the phosphorylation level at Tyr705 (p-STAT3). As shown in Figure 1F, SC99 was the most potent one to inhibit STAT3 phosphorylation in both cell lines examined. JP31 also inhibited STAT3 activation but less potent than SC99 which was probably due to the 3-chloro-4-fluorophenyl group, especially the chlorine and fluorine atoms that were missing from JP31.

### SC99 inhibits STAT3 activation and dimerization

**Figure 2:**
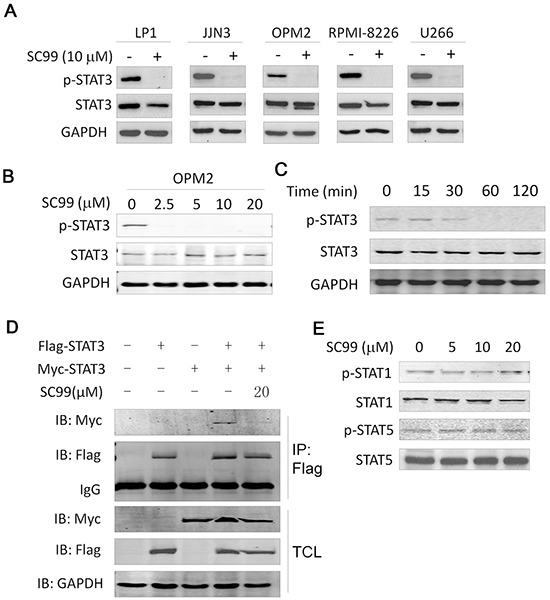
SC99 inhibits STAT3 activation in MM cells **A.** Five MM cell lines were treated with or without 10 μM of SC99 overnight followed by cell lysate preparation and immunoblotting analysis for p-STAT3 (Tyr705) and total STAT3. **B.** OPM2 cells were treated with SC99 at the indicated concentrations followed by the analysis of the expression of p-STAT3. **C.** OPM2 cells were treated with SC99 (10 μM) at indicated time periods. Expression of p-STAT3 and STAT3 was measured by immunoblotting assay. D, HEK293T cells were transfected with Myc- and/ or Flag-STAT3 for 40 hrs, followed by SC99 treatment for 8 hrs. Cell lysates were then prepared for immunoprecipitation (IP) with an anti-Flag antibody and subsqeuent immunoblotting. TCL: total cell lysates. **E.** OPM2 cells were treated with SC99 for 24 hrs followed by immunoblotting with specific antibodies against STAT1, p-STAT1, STAT5 and p-STAT5.

To investigate the effect of SC99 on the constitutive activation of STAT3, five MM cell lines with highly activated STAT3 were treated with SC99 (10 μM) or DMSO, followed by immunoblotting analyses against p-STAT3. As shown in Figure 2A, SC99 decreased the p-STAT3 level but had no effects on total STAT3 expression in all cell lines examined. Moreover, SC99 was found to suppress STAT3 activation in a concentration- and time course-dependent manner (Figure 2B and 2C). STAT3 phosphorylation was decreased at a concentration as low as 2.5 μM of SC99, and it was completely inhibited by SC99 at 10 μM (Figure 2B). In the time-course study, constitutive STAT3 phosphorylation was suppressed by SC99 within 30 min in OPM2 cells (Figure 2C). Therefore, SC99 was potent to inhibit STAT3 activation.

STAT3 activation leads to STAT3 dimerization and further is translocated to the nuclei where it exerts its transcriptional activity [22]. To evaluate whether SC99 interferes with STAT3 dimerization, we designed a Myc- and a Flag-tagged STAT3 plasmid. These two plasmids were then co-transfected into HEK293T cells followed by SC99 treatment. The whole cell lysates were then subjected to immunoprecipitation/immunoblotting assay. As shown in Figure 2D, dimerization of Myc-STAT3:Flag-STAT3 was found in the absence of SC99, but this dimerization was significantly decreased when SC99 was added. Because STAT3 dimerization is an outcome of STAT3 activation, this experiment further demonstrated that SC99 inhibited STAT3 activation.

There are 7 members in the STAT3 family, of which STAT1, STAT3 and STAT5 are associated with oncogenesis, to exclude the effects of SC99 on STAT1 and STAT5, MM cells were treated with SC99 for 24 hrs followed by immunoblotting analysis. It turned out that SC99 dose-dependently suppressed STAT3 phosphorylation, but had no effects on the p-STAT1 or p-STAT5 level (Figure 2E). Therefore, SC99 preferred to inhibit STAT3 activation.

### SC99 inhibits STAT3 nuclear translocation

**Figure 3:**
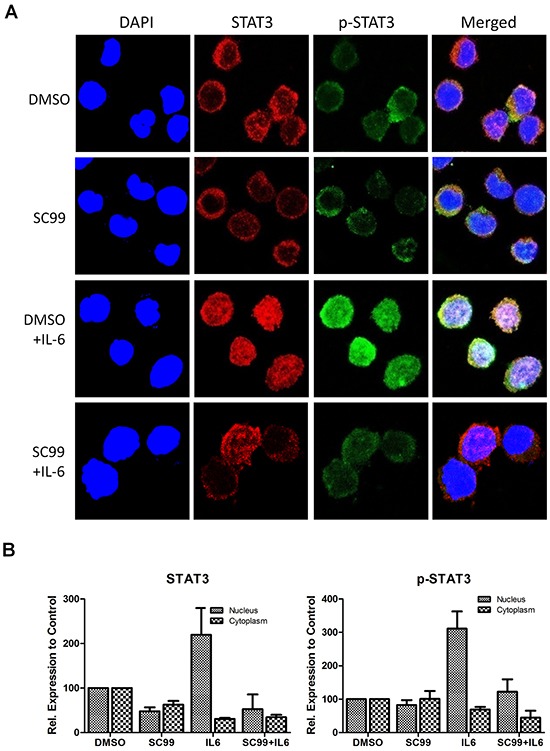
SC99 inhibits STAT3 nuclear translocation **A.** OPM2 cells were starved overnight followed by treatment with SC99 or DMSO for 2 hrs. Cells were then treated with 50 ng/ml of IL-6 for 20 min. For immunofluorescent assay, cells were fixed and stained with anti-phospho-STAT3 (p-STAT3) and DAPI before subject to confocal microscopy analysis. Red, total STAT3; Green, p-STAT3; blue, nuclei. **B.** Statistical analysis of STAT3 and p-STAT3 subcellular distribution using Image J software.

STAT3 is a transcription factor and it must enter nuclei to exert its activity after its activation. The above experiments showed that SC99 potently inhibited STAT3 activation, to further evaluate whether SC99 affected STAT3 nuclear translocation, an immunofluorescent assay was performed. Starved OPM2 cells were pre-treated with DMSO or SC99 followed by IL-6 stimulation for 20 min because IL-6 can trigger STAT3 phosphorylation and nuclear import [23]. The immunofluorescent assay revealed that in the resting status without IL-6 stimulation, both STAT3 and p-STAT3 were distributed around the nuclei or in the cytosol, however, when IL-6 was added, both total and activated STAT3 were mainly found in the nuclei (Figure 3A and 3B). As expected, pretreatment of SC99 prevented STAT3 from activation and nuclear translocation, which was similar to a previous report [23]. These findings further demonstrated that SC99 inhibited STAT3 activation.

### SC99 inhibits JAK2 but not other kinases associated with STAT3 signaling

**Figure 4:**
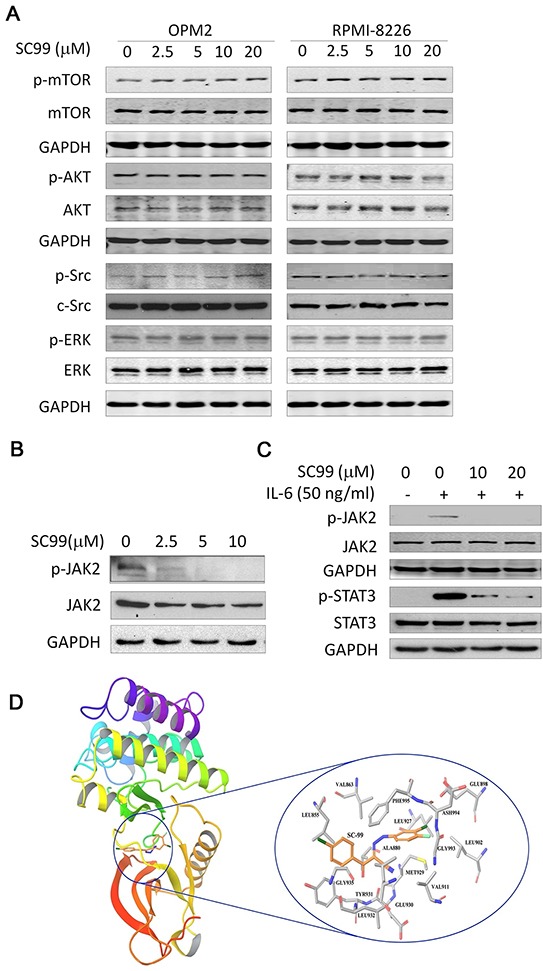
SC99 inhibits JAK2 but not other associated kinases **A.** OPM2 and RPMI-8226 cells were treated with SC99 for 60 min at indicated concentrations, followed by immunoblotting assays for the phosphorylation levels of mTOR, AKT, ERK and c-Src. **B.** OPM2 cells were treated with SC99 followed by JAK2 activation. **C.** OPM2 cells were starved overnight followed by SC99 treatment. Sixty minutes later, cells were stimulated by IL-6 for 15 min. Cell lysates were prepared for immunoblotting analysis to measure the expression level of p-JAK2 and p-STAT3. JAK2, STAT3 and GAPDH were used as controls. **D.** SC99 interacts with JAK2 active pocket analyzed by computer modeling using the *Glide* module in the Schrodinger.

Several important signaling pathways are in association with STAT3 activation, including MAPK/ERK [24], PI3K/AKT/mTOR [25], c-Src [26], and JAK pathways [24]. We next evaluated the effects of SC99 on these specific kinases including JAK2, ERK, AKT, mTOR, and c-Src by immunoblotting. As shown in Figure 4A, although SC99 steadily suppressed STAT3 activation, it did not inhibit the phosphorylation levels of AKT, ERK, mTOR or c-Src at a concentration up to 20 μM, which suggested that the inhibition of SC99 on STAT3 was not an off-target effect.

JAK2 is a key kinase activating STAT3, we therefore evaluated the effect of SC99 on JAK2. As shown in Figure 4B, SC99 inhibited JAK2 phosphorylation in a concentration-dependent manner. At 2.5 μM, SC99 was capable to inactivate JAK2 in a manner similar to the inhibition on STAT3 (Figure 2B). As reported previously, STAT3 can be activated by the cytockine IL-6 via JAK2 signaling [1, 2]. To find out whether SC99 was able to repress IL-6-stimulated JAK2 and STAT3 activation, starved OPM2 cells were pre-treated with SC99 followed by IL-6 stimulation. As expected, addition of IL-6 raised the phosphorylation level of both JAK2 and STAT3 which could be suppressed by SC99 within 30 min (Figure 4C).

To further evaluate whether SC99 could interfere with the JAK2 molecule, a computer modeling was performed. The results showed that SC99 was well docked into the ATP-binding pocket of JAK2 in which SC99 formed strong and stable hydrogen bond (red line in Figure 4D) with Leu932 of JAK2. The H-bond could prevent SC99 from escaping from the active sites and enhanced the inhibition of JAK2. Moreover, because of the hydrophobicity of the ATP-pocket, SC99 formed extensive van der Waals interactions with the hydrophobic residues of JAK2 (Figure 4D). These results further indicated that SC99 strongly interacted with JAK2 and led to the inhibition of JAK2.

### SC99 downregulates STAT3-modulated gene expression

**Figure 5:**
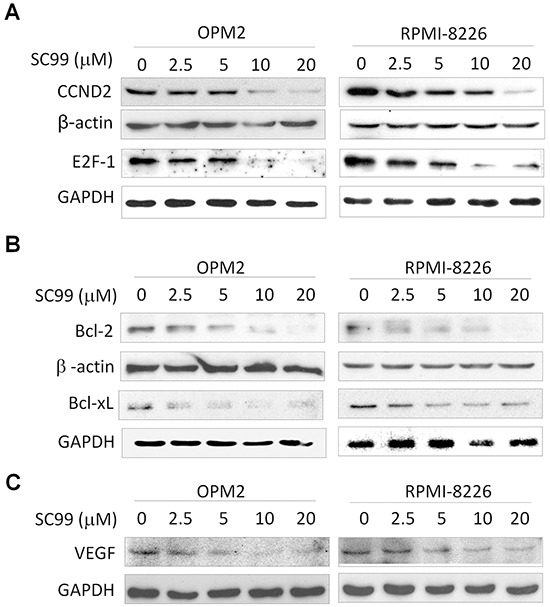
SC99 downregulates STAT3-regulated genes in MM cells OPM2 and RPMI-8226 cells were treated with increasing concentrations of SC99 for 24 hrs. After incubation, cells were harvested and total proteins were isolated for immunoblotting analysis against CCND2 and E2F-1 **A.**, Bcl-2 and Bcl-xL **B.** and VEGF **C.** GAPDH and β-actin were used as loading control.

As a key transcription factor, STAT3 modulates the expression of a panel of key cell cycle regulators such as *cyclins* and transcription factor *E2F-1* [7, 9]. Because STAT3 inhibitors usually result in decreased expression of STAT3 targeted genes [27], and the above studies had demonstrated that SC99 was a JAK2/STAT3 inhibitor. We subsequently evaluated the effect of SC99 inhibition on STAT3 downstream gene expression by immunoblotting. As shown in Figure 5A, SC99 suppressed the expression of cyclin D2 and E2F-1 in a concentration-dependent manner which was similar to the effects of SC99 on the promoter transactivation of cyclin D2 as shown in Figure 1A. Because these proteins are key regulators of cell cycle progression, SC99 arrested MM cells at the G1 phase (Supplementary Figure S1).

STAT3 is also involved in cell survival by upregulating several important genes, such as *Bcl-2* and *Bcl-xl* [4, 5]. To further view the effects of SC99 on the activity of STAT3, we next measured the protein levels of Bcl-2 and Bcl-xl. As shown in Figure 5B, both Bcl-2 and Bcl-xl was decreased by SC99 in a concentration-dependent manner (Figure 5B). SC99 also downregulated the expression of *VEGF,* another STAT3 substrate gene (Figure 5C). All of these results thus further demonstrated SC99 inhibited STAT3 activity.

### SC99 induces MM cell apoptosis

**Figure 6:**
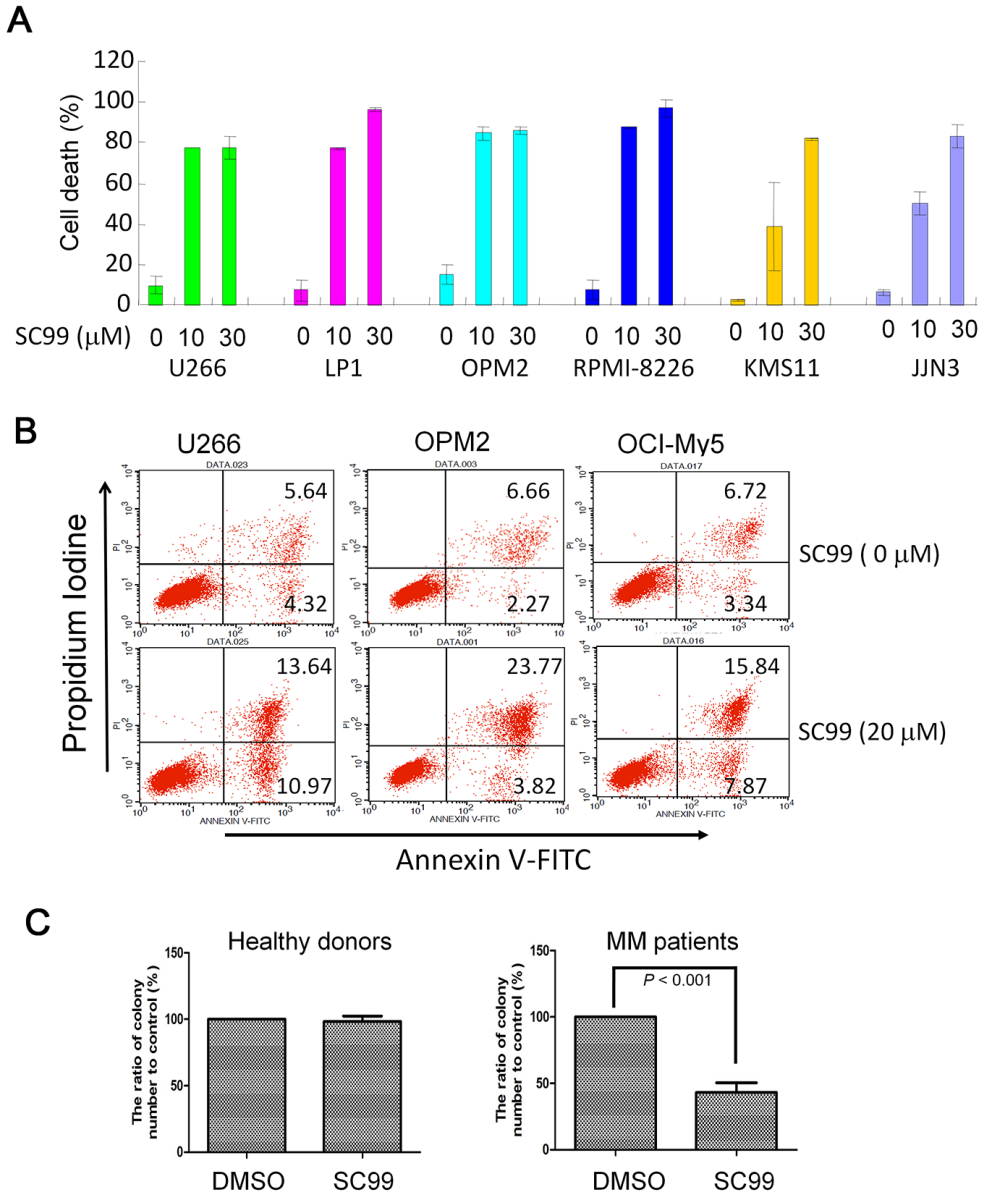
SC99 induces MM cell death **A.** Six MM cell lines were treated with SC99 (10 or 30 μM) for 72 hrs, followed by trypan blue exclusion assay. **B.** U266, OPM2 and OCI-MY5 were treated with 20 μM of SC99 for 24 hrs. Cells were then stained with Annexin V-FITC and PI and analyzed on a BD FACSCalibur™ flow cytometer. **C.** mononuclear cells were isolated from bone marrow collected from healthy donors or MM patients. These cells were then treated with SC99 (10 μM) for 10 hrs before being plated in triplicate in MethoCult GF H4434 medium.

Because JAK2-STAT3 signaling pathway is critical for MM cell survival, we next evaluated SC99-induced cell death by Trypan blue exclusion and flow cytometry. As shown in Figure 6A, SC99 induced most of MM cell death within 72 hrs at 10 μM. A more precise assay with Annexin V staining, a benchmark of early apoptosis, was performed. As shown in Figure 6B, more than 20% of cells underwent apoptosis after treatment with SC99 for 24 hrs. To further evaluate whether SC99 was effective in primary MM cells, a colony forming assay was performed in bone marrow cells from both healthy donors and MM patients. As shown in Figure 6C, SC99 had no effects on colony forming from healthy bone marrow cells, but markedly suppressed colony forming in MM patients. These results demonstrated that SC99 was toxic to MM cells and it might affect MM cell self-renewal.

### SC99 activates apoptotic signaling but it is attenuated by overexpressed STAT3

**Figure 7:**
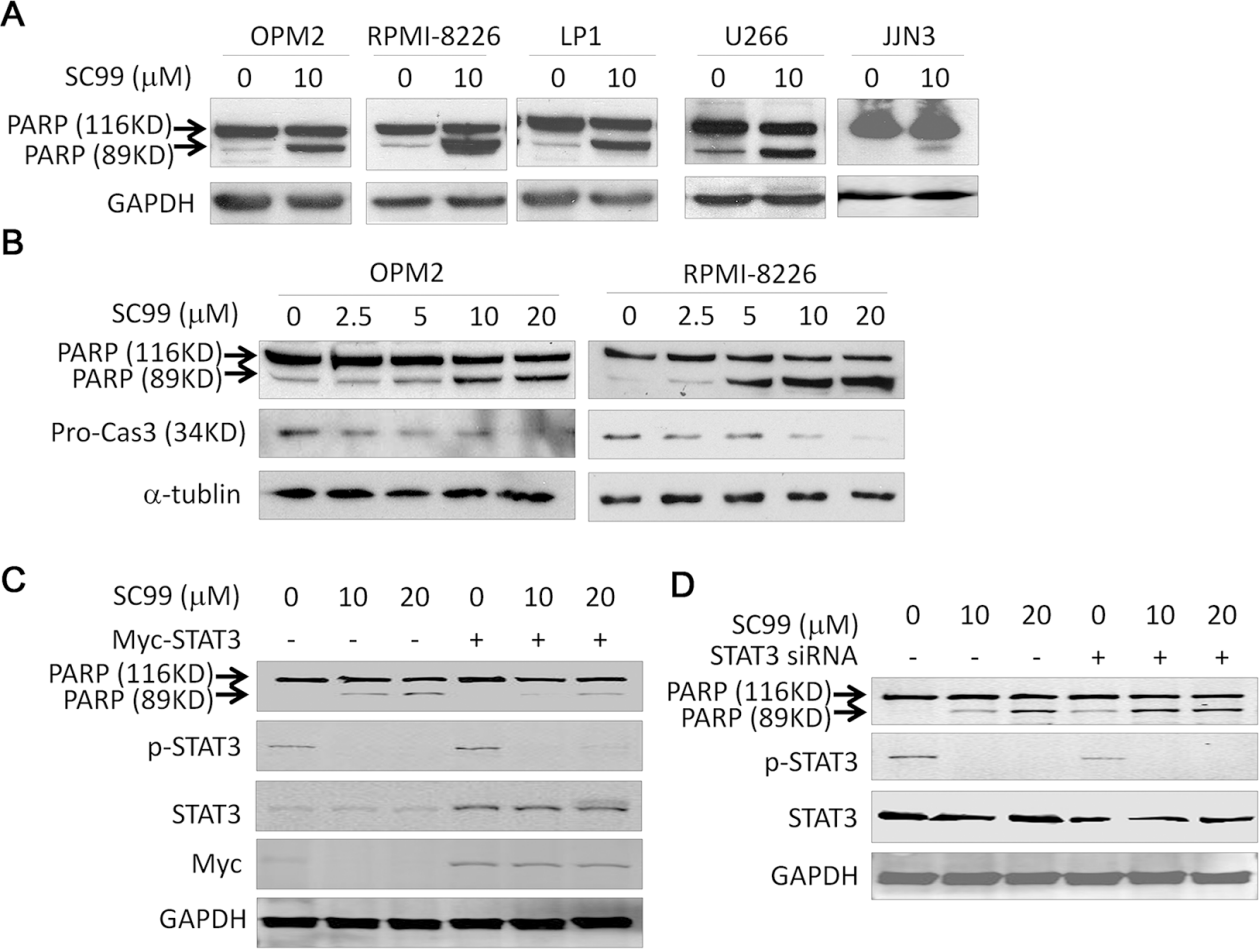
SC99 activates apoptotic signaling pathway but it is attenuated by ectopic STAT3 **A.** MM cell lines were treated with 10 μM SC99 or DMSO for 24 hrs followed by lysate preparation and immunoblotting analysis against PARP and GAPDH. **B.** OPM2 and RPMI-8226 cells were treated with SC99 at indicated concentrations for 24 hrs followed by analysis for PARP and Caspase-3. GAPDH and α-tublin were used as a loading control. **C.** OPM2 cells were trasnfected with a STAT3 plasmid for 24 hrs followed by SC99 treatment for another 24 hrs. Cells were then applied for immunoblotting assay against specific proteins as indicated. **D.** HeLa cells were transfected with a STAT3 siRNA for 24 hrs, followed by SC99 treatment for another 24 hrs. Cells were than subject to immunoblotting assay against specific proteins as indicated.

To further evaluate SC99-induced cell apoptosis, we evaluated PARP and caspase-3, two representative hallmarks of cell apoptosis, in MM cells treated with SC99. PARP was cleaved in all MM cell lines examined (Figure 7A), and this cleavage was concentration-dependent, in a manner similar to caspase-3 cleavage (Figure 7B). These results indicated that cell apoptotic signaling was activated by SC99. Because STAT3 was demonstrated to be the target of SC99, we next questioned whether the ectopic expression of STAT3 could rescue cell death induced by SC99. OPM2 cells were transfected a STAT3 plasmid before being treated with SC99. Immunoblotting showed that SC99 induced a marked cleavage of PARP in the cells which was partly abolished by ectopic expression of STAT3 (Figure 7C). We also evaluated cell death when STAT3 was knocked down. STAT3 siRNA was infected into HeLa cells, 24 hrs later, cells were treated with SC99 for another 24 hrs. As shown in Figure 7D, STAT3 knockdown could slightly induce PARP cleavage because STAT3 was still expressed at a certain level, but cell death was markedly enhanced by SC99 at which p-STAT3 was fully suppressed (Figure 7D). Taken together, all these results further demonstrated that SC99 induced cell apoptosis by targeting STAT3.

### SC99 delays tumor growth in MM xenograft models

**Figure 8:**
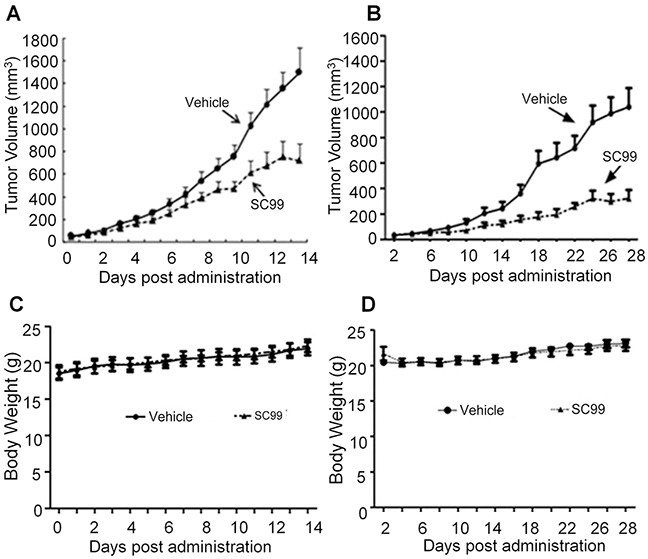
SC99 delays myeloma tumor growth in xenograft mice models Human MM cells OPM2 **A.** or JJN3 **B.** were injected subcutaneously into nude mice with a density of 30 million cells/site/mouse. When tumors were palpable, mice (*n* = 10/group) were orally given SC99 (30 mg/Kg body weight) in PBS containing 10% Tween 80 and 10% DMSO daily for continuous 14 or 28 days. Tumor volumes (A and B) and mouse body weight **C.** and **D.** were monitored every day or every other day.

To further evaluate the anti-myeloma efficacy of SC99 *in vivo*, two independent human myeloma xenograft models were established. One was established by *s.c.* inoculation of JJN3 cells into nude mice because compared with other MM cell lines, JJN3 was more adherent and resistant to several drugs such as dexamethasone [21] and it was easier to grow into a tumor in nude mice. Oral administration of SC99 (30 mg/kg body weight every day) led to a marked delay in tumor growth in 7 days (*p*<0.05). On the 14th day, SC99 significantly slowed down tumor growth with a *p* value < 0.01 (Figure 8A). To confirm this finding, another MM xenograft model was established with OPM2 cells. The same treatment showed that SC99 suppressed tumor growth more than 40% in 14 days in the OPM2 model (Figure 8B), which was similar to that observed in the JJN3 model. However, there were no significant differences in the body weight over the experimental period in both models (Figure 8C and 8D).

## DISCUSSION

In the above study, we identified a JAK2/STAT3 inhibitor that displays potent activity against MM in both cellular and animal models.

Several key steps are involved in the modulation of STAT3 activation. Upon stimulation, STAT3 is rapidly phosphorylated at Tyr705 and forms a homo- or hetero-dimer which subsequently translocates to the nuclei where it binds to target genes and modulates their transcription. For example, BP-1-102, a recently reported STAT3 inhibitor, inhibits STAT3 activity by binding to the SH2 domain which is critical for the formation of the STAT3 dimers through reciprocal phosphotyrosine–SH2–binding interactions [28]. S3I-1757, another small molecule also inhibits STAT3 activity by this manner [29]. In our study, SC99 inhibit STAT3 activation and dimerization, which leads to decreased p-STAT3 level and nuclear import as evidence of immunofluorescent assay. However, different from BP-1-102 and S3I-1757, SC99-induced STAT3 inhibition is probably due to the inhibition of JAK2 activity. JAK2 is located at the upstream of STAT3 signaling pathway and is the major activator of STAT3. Our study confirmed this hypothesis because SC99 inhibits JAK2 activation but not other closely related kinases, such as AKT, ERK and c-Src, which is similar to previous studies [28]. Moreover, computer modeling also found that SC99 is well fitted into the active pocket of JAK2 and interacts with key amino acid residues thus interfering with its activity. Therefore, SC99 displays potent inhibition on STAT3 activation probably via the JAK2 kinase and SC99 is a JAK2/STAT3 inhibitor.

As a critical transcription factor, STAT3 modulates the transcription and expression of a broad panel of important genes, including angiogenic *VEGF*, pro-survival *Bcl-2*, *Mcl-1*, anti-apoptotic *survivin* and *XIAP*, as well as cell cycle regulators, including cyclins, cyclin-dependent kinase and transcription factor *E2F-1*. Our study demonstrated that SC99 suppresses these gene expressions. Importantly, these genes are also involved in MM cell proliferation and progression and chemoresistance [13, 30]. Bcl-2 is over-expressed in MM cells and plays a “gatekeeper” role in control of the integrity of mitochondrial membrane and blocks the mitochondria-derived cell apoptosis [31]. D-type cyclins are universally dysregulated in MM cells, and confer to chemoresistance and poor prognosis [18, 32, 33]. By inhibiting STAT3 activation and cell cycle regulators, SC99 thus decreases proliferation and increases apoptosis of MM cells. Moreover, SC99-activated apoptosis could be partly abolished by ectopic expression of STAT3, further suggests STAT3 is the target of SC99.

Safety is a critical factor in drug development, many candidates are abandoned because of the safety issue. As a critical transcription factor, STAT3 is also important for normal biological activity in addition to cancer cells. Disruption of STAT3 therefore is potentially toxic to normal cells. However, our animal studies showed SC99 is not toxic but affects tumor growth. The cellular studies also showed that SC99 prefers to suppress myeloma cell growth and proliferation but does not affect normal bone marrow cells. Therefore, SC99 is a relatively safe candidate for myeloma therapy.

In summary, the present study identified a novel JAK2/STAT3 inhibitor. Given its safety and efficacy in vitro and in vivo, SC99 could be developed as a promising oral active anti-myeloma drug, however, further extended safety and therapeutic efficacy evaluation must be performed.

## MATERIALS AND METHODS

### Cell culture

MM cell lines LP1 and JJN3 were kindly provided by Dr. Aaron Schimmer from Ontario Cancer Institute, Toronto, Canada. MM cell lines RPMI-8226, OPM2, and U266, and HeLa were purchased from American Type Culture Collection. All cells were grown in Iscove's Modified Dulbecco's Medium supplemented with 10% fetal bovine serum (Hyclone), penicillin (100 units/mL) and streptomycin (100 μg/mL) in an incubator humidified with 95% air and 5% CO_2_ at 37°C.

Primary bone marrow samples were collected from the Department of Hematology, the First Affiliated Hospital of Soochow University, with the approval from the Institutional Review Board of Soochow University. Informed consent was obtained in accordance with the Declaration of Helsinki. Mono-nuclear cells were isolated by Lympholyte® Cell Separation (Cedarlane, Canada) [34].

### Constructs of luciferase reporters

A STAT3 luciferase construct (p-STAT3-luc) driven by a quadrupled STAT3 response element was obtained from Beyotime Biotechnology Institute (Nantong, China). A cyclin D2 luciferase reporter plasmid was established in pGL4 vector (Promega) driven by a fragment of cyclin D2 promoter containing or lacking a STAT3 response element [21]. A NF-κB luciferase reporter construct (pNF-κB-Luc) was provided by Clontech Laboratories.

### Identification of inhibitors of cyclin D2 transactivation

The plasmid p-STAT3.Luc was stably introduced into NIH3T3 cells which was used to screen Maybridge Chemicals (Trevillett, UK) using a protocol as described previously under the robot assistance [21, 34, 35]. Compounds downregulated CCND2-luciferase activity to 50% or higher but did not induce 90% of viable after treatment were chosen for further studies [21].

### Preparation of SC99 and analogs

SC99 is chemically named 2-(2-(3-chloro-4-fluorophenyl)hydrazono)-3-(4-chlorophenyl)-3-oxo-pro panenitrile. SC99 and its analogs were synthesized from 4-(trifluoro-methyl)benzoamine and 2-(2-thiopheneSulfonyl) acetonitrile. The detailed method was available in Supplementary File 1. All chemicals were confirmed by NMR and Mass spectrometry.

### Luciferase assay

Specific luciferase constructs were transiently introduced into HEK293 cells using 25 KD polyethylenimine (Sigma, St. Louis, MO) as described previously [36]. Luciferase activity evaluation was performed using a Bright-Glo luciferase assay system (Promega) [21].

### Flow cytometry

To determine cell apoptosis, MM cells treated with SC99 were stained with Annexin V-fluorescein isothiocyanate (Annexin V-FITC) and propidium iodide (PI, Sigma) according to the manufacturer's instruction. Stained cells were analyzed on a flow cytometer (FACSCalibur®, Becton Dickinson). To determine cell cycle, MM cells after SC99 treatment was stained with PI and subsequent analysis on the flow cytometer as described previously [34].

### Cloning of the STAT3 gene

To clone *STAT3*, total RNA was prepared from U266 cells and used for the synthesis of the first strand cDNA in a cDNA reaction system (TransGen Biotech, Beijing, China). The *STAT3* gene was then cloned into a pcDNA3.1 vector with a Myc or a Flag tag using primers: 5′-CCCAAGCTTATGGCCCAATGGAATCAGCTACAG -3′ (Forward primer) and 5′-CATCTCGAGTCACATGG GGGAGGTAGCGCACT-3′ (Reverse primer). STAT3 siRNA was designed and provided by Genepharm Co. Ltd, Suzhou, China.

### Molecular docking

The structure of SC99 was constructed in Schrodinger (version 09) and then preprocessed with the *LigPrep* module. The structure of JAK2 (PDB ID: 3UGC) was used as the initial structure. All crystallographic water molecules were removed, hydrogen atoms were added, and the structure was submitted to restrained minimization to relieve steric clashes using the OPLS2005 force field within the *Protein Preparation Wizard* in Schrodinger. The minimization was terminated when the root-mean-square deviation (RMSD) reached a maximum value of 0.3 Å. For the grid generation and ligand docking procedures, the default settings were used and the *Glide* module in the Schrodinger was employed for molecular docking [37–39].

### Immunoblotting

Cells after SC99 treatment were prepared for immunoblotting according to our previous method [35, 40]. A specific primary antibody against cyclin D_2_ (CCND2) was purchased from Santa Cruz Biotechnology, Inc. (Santa Cruz, CA); antibodies against Bcl-2, Bcl-xL, Caspase-3, STAT3, p-STAT3(Tyr705), c-Src, p-Src, JAK2, p-JAK2, E2F-1, AKT, p-AKT, ERK, p-ERK, mTOR, p-mTOR, and PARP were purchased from Cell Signaling Technology (Danvers, MA). Antibodies against VEGF, β-actin, α-tubulin, anti–mouse immunoglobulin G (IgG) and anti–rabbit IgG horseradish peroxidase conjugated antibody were purchased from R&D Systems (Minneapolis, MN). Anti-Myc antibody and GAPDH was purchased from Sigma (St. Louis, MO).

### Immunofluorescence staining

The basic protocol for the analysis of p-STAT3 subcellular location was referred to our previous protocol [35]. Briefly, OPM2 cells were starved overnight, followed by incubation with SC99 or DMSO for 60 min. Cells were then stimulated with 50 ng/ml of IL-6 for 20 min before being harvested in Tris-buffered saline (TBS) containing 1 mM sodium orthovanadate. Cells were transferred to polylysine-coated slides by cytospin and dried in air for 20 min at room temperature. After being fixed in 2% of paraformaldehyde for 10 min and permeabilized in 0.1% Triton-100 for 5 min, cells were blocked in TBS containing 5% bovine serum albumin for 1 hr at room temperature before being stained with STAT3 or p-STAT3 (Tyr705) overnight at 4°C. Then cells were gently washed and incubated with FITC conjugated goat anti-rabbit IgG (Beyotime Biotechnology Ltd). Finally, slides were incubated with 5 μg/ml of 4′,6-diamidino-2-phenylindole (DAPI, Beyotime) for 10 min. The subcellular location and relative abundance of STAT3 or p-STAT3 were immediately analyzed with an Olympus confocal microscope.

### Colony forming assay

To assess clonogenic growth in bone marrow cells (6.25 × 10^5^/mL) from primary MM patients or healthy donors, a colony forming unit assay was performed as described previously [34]. Cells treated with SC99 or buffer control for 24 hrs were washed and equal volumes were plated in triplicate in MethoCult GF H4434 medium (StemCell Technologies, Vancouver, BC) containing 1% methycellulose in IMDM, 30% FCS, 1% bovine serum albumin, 3 U/mL of recombinant human erythropoietin, 0.1 mM of 2-mercaptoethanol, 2 mM of L-glutamine, 50 ng/mL of recombinant human stem cell factor, 10 ng/mL of GM-CSF, and 10 ng/mL of rh IL-3. Seven days (AML samples) or 14 days (normal PBCS) after plating, the number of colonies containing 20 or more cells was counted as described previously [34].

### Myeloma xenograft study

Two MM xenograft models were established with human MM cell line JJN3 and OPM2, respectively, as described previously [35]. Thirty million of MM cells were injected subcutaneously in the right flanks of each nude mouse (5-6 weeks old, female, Shanghai Slac Laboratory Animal Co. Ltd.). When tumors were palpable, mice were randomly divided into two groups, one was given SC99 (30 mg/kg body weight) in PBS containing 10% Tween 80 and 10% DMSO daily, another group was received the vehicle only. Tumor sizes and body weight were monitored every day [34]. This xenograft study was approved by the Review Board on Experimental Animals of Soochow University.

### Statistical analysis

Statistical significance of differences observed in drug-treated *vs.* control cells or animals was determined by using the Student's *t* test. The minimal level of significance was *P* < 0.05.

## SUPPLEMENTARY FIGURE


