# Correction: A novel small molecule agent displays potent anti-myeloma activity by inhibiting the JAK2-STAT3 signaling pathway

**DOI:** 10.18632/oncotarget.13803

**Published:** 2016-12-06

**Authors:** Zubin Zhang, Hongwu Mao, Xiaolin Du, Jingyu Zhu, Yujia Xu, Siyu Wang, Xin Xu, Peng Ji, Yang Yu, Biyin Cao, Kunkun Han, Tingjun Hou, Zhuan Xu, Yan Kong, Gaofeng Jiang, Xiaowen Tang, Chunhua Qiao, Xinliang Mao

**Present**: Due to an error made during the final figure assembly, blots for U266 were mistakenly used in lieu of blots for JJN3 in Figure 2A.

**Correct**: Corrected Figure 2 is provided below. The authors sincerely apologize for this oversight.

Original article: Oncotarget. 2016; 7(8):9296-308. doi: 10.18632/oncotarget.6974.

**Figure 2:**
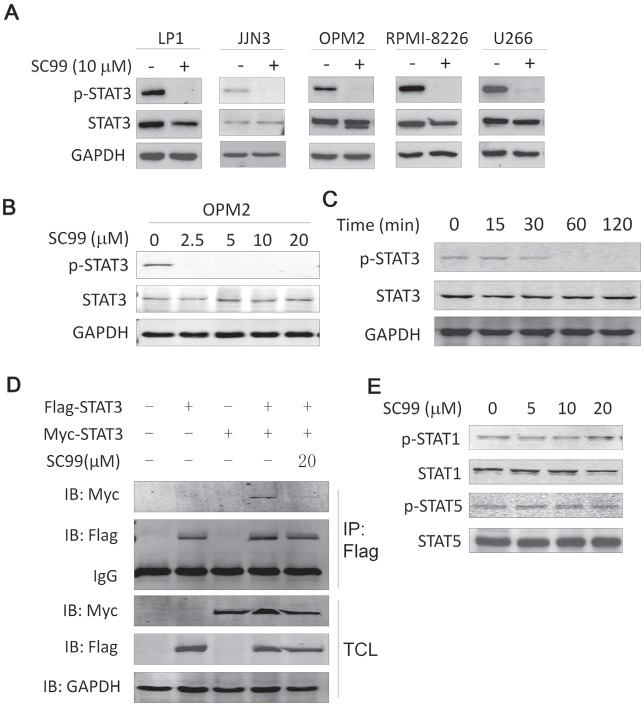
SC99 inhibits STAT3 activation in MM cells **A**. Five MM cell lines were treated with or without 10 μM of SC99 overnight followed by cell lysate preparation and immunoblotting analysis for p-STAT3 (Tyr705) and total STAT3. **B**. OPM2 cells were treated with SC99 at the indicated concentrations followed by the analysis of the expression of p-STAT3. **C**. OPM2 cells were treated with SC99 (10 μM) at indicated time periods. Expression of p-STAT3 and STAT3 was measured by immunoblotting assay. **D**, HEK293T cells were transfected with Myc- and/ or Flag-STAT3 for 40 hrs, followed by SC99 treatment for 8 hrs. Cell lysates were then prepared for immunoprecipitation (IP) with an anti-Flag antibody and subsqeuent immunoblotting. TCL: total cell lysates. **E**. OPM2 cells were treated with SC99 for 24 hrs followed by immunoblotting with specific antibodies against STAT1, p-STAT1, STAT5 and p-STAT5.

