# Supplementary material for: A novel small molecule agent displays potent anti-myeloma activity by inhibiting the JAK2-STAT3 signaling pathway
Source: Oncotarget. 2016 Jan 22;7(8):9296–308. doi: 10.18632/oncotarget.6974 (PMC4891041; doi:10.18632/oncotarget.6974)
Supplement: Supplementary file 1 [file oncotarget-07-9296-s001.pdf]

SYNTHESIS OF SC99 AND ITS ANALOGS

General

All reagents and solvents were of reagent grade or purified according to standard methods. Analytical thin layer chromatography (TLC) was performed with silica gel plates using silica gel 60 GF_{254} (Qingdao Haiyang Chemical Co., Ltd.). Column chromatography was performed over silica gel (200–300 mesh, Qingdao Marine Chemical Ltd, P. R. China). The ^{1}H NMR and ^{13}C NMR spectra were recorded in deuterochloroform at ambient temperature using a Varian Mercury 400. The ESIMS were recorded with an Agilent 1260LC. PE: petroleum ether (bp: 60–90°C); EA: ethyl acetate; DCM: dichloromethane; THF: tetrahydrofuran.

Scheme 1: Synthetic route for the designed Stat3 inhibitors

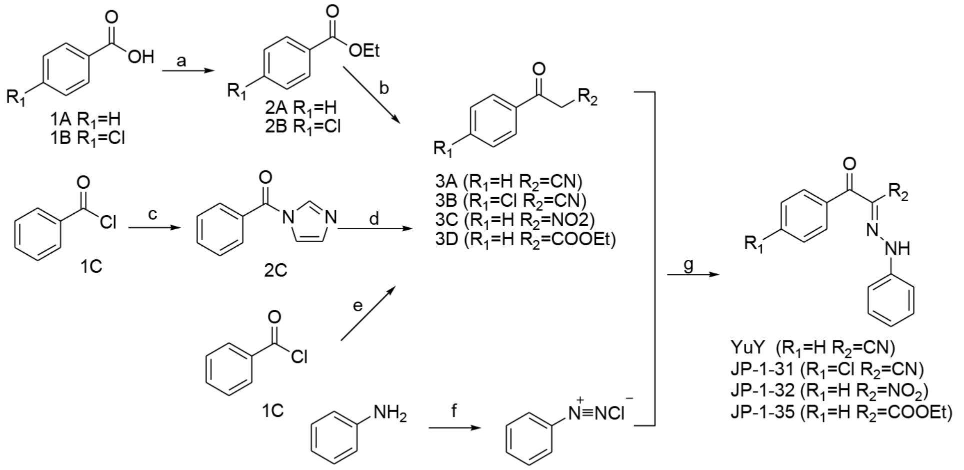

Reagents and conditions: (a) EtOH, $SOCl_2$, 78 °C, 2 h; (b) CH_3CN, $NaOCH_3$, 78 °C, 3 h; (c) Imidazole, Et_3N, DCM, 0.5 h; (d) CH_3NO_2, NaH, 10 °C to rt, 13 h; (e) NaH, ethyl acetate, THF, 0 °C, 2 h; (f) HCl, $NaNO_2$, 0 °C, 5 min; (g) NaOAc, MeOH, H_2O, rt, 1 h.

Ethyl benzoate (2A)

The benzoic acid (1.0 g, 8.19 mmol) was dissolved in EtOH (10 mL). $SOCl_2$ (1.46 g, 12.27 mmol) was slowly added to the solution with vigrous stirring. The mixture was then warmed to 78 °C and refluxed for 2 hours. Upon the completion of the reaction, the solution was concentrated under reduced pressure to remove excess EtOH and $SOCl_2$. The residue was then diluted with EA (50 ml) and washed twice with H_2O, saturated $NaHCO_3$ solution and brine. The organic phase was dried over anhydrous Na_2SO_4, concentrated to give the product as a colorless oil (1.20 g, 98%).^{1}H NMR (400 MHz, $CDCl_3$) δ 8.02 (dd, J = 5.9, 2.1 Hz, 2H), 7.50 (dd, J = 9.2, 4.3 Hz, 1H), 7.38 (dd, J = 10.9, 4.0 Hz, 2H), 4.34 (td, J = 6.6, 1.6 Hz, 2H), 1.35 (dd, J = 9.2, 4.8 Hz, 3H).

Ethyl 4-chlorobenzoate (2B)

Compound 2B was prepared from the corresponding 4-chlorobenzoic acid using the procedure described for 2A in 97% yield as a colorless oil, which was used for subsequent reaction without further purification.

3-oxo-3-phenylpropanenitrile (3A)

A mixture of ethyl benzoate (158 mg, 1.05 mmol), sodium methoxide (97 mg, 1.79 mmol) in acetonitrile (5 mL) was refluxed for 3 h. After cooling to room temperature, formation of white precipitate was observed, which was filtered and redissolved in water (5 mL). 2M HCl (1.5 mL) was added to the solution and the mixture was extracted with DCM (10 mL × 2). The combined organic layers were washed with brine and dried over anhydrous Na_2SO_4. The solvent was removed under reduced pressure to give the crude product, which was purified by flash chromatography (petroleum ether/ethyl acetate 3:1) to provide pure 3-oxo-3-phenylpropanenitrile *(3A)* as a white solid (89 mg, 58%).^{1}H NMR (400 MHz, $CDCl_3$) δ 7.91 (d, J = 7.2 Hz, 2H), 7.66 (t, J = 7.3 Hz, 1H), 7.52 (t, J = 6.9 Hz, 2H), 4.10 (s, 2H).

3-(4-chlorophenyl)-3-oxopropanenitrile (3B)

Compound 3B was prepared from the ethyl 4-chlorobenzoate using the procedure described for 2A in 61% yield as a white solid. ^{1}H NMR (400 MHz, $CDCl_3$) δ 7.86 (d, J = 8.5 Hz, 2H), 7.50 (d, J = 7.8 Hz, 2H), 4.09 (d, J = 0.5 Hz, 2H).

N-acylimidazole (2C)

Imidazole (408.5 mg, 6 mmol) and triethylamine (607.1 mg, 6 mmol) were dissolved in dry DCM (25 mL), and acyl chloride (702.8 mg, 5 mmol) was slowly added to the solution. The reaction mixture was stirred for 0.5 h at room temperature. The mixture was filtered, and the solution was rapidly washed with cold water (150 mL × 3). The organic phase was dried over anhydrous Na_2SO_4 and concentrated, yielding the crude N-acylimidazole, which was used for the synthesis of 2-nitro-1-phenylethanone without further purification.

2-nitro-1-phenylethanone (3C)

A mixture of the nitromethane (390 mg, 6.4 mmol) and sodium hydride (337 mg, 11 mmol) in DMSO (25 mL) was stirred for 0.5 h at 10 °C. The crude N-acylimidazole (1.1 g, 6.4 mmol) in DMSO (25 mL) was added dropwise to the resulting solution and the mixture was stirred for 30 minutes at 10 °C and then at room temperature for 12 h. The mixture was diluted with ethyl acetate (150 mL), poured into 2 M HCl (150 mL), and then extracted with ethyl acetate (150 mL × 3). The combined organic extracts were washed with cold water (150 mL × 3), dried over anhydrous Na_2SO_4, filtered, and concentrated. The residue was purified by flash chromatography (petroleum ether/ethyl acetate 1:1) to give the corresponding 2-nitro-1-phenylethanone as a white solid (971 mg 76%).^{1}H NMR (400 MHz, $CDCl_3$) δ 7.87 (d, *J* = 7.7 Hz, 2H), 7.69 (t, *J* = 7.3 Hz, 1H), 7.54 (t, *J* = 7.4 Hz, 2H), 5.91 (s, 2H).

Ethyl 3-oxo-3-phenylpropanoate (3D)

To the solution of benzoyl chloride (500 mg, 3.55 mmol) and EA (344.7 mg, 3.91 mmol) in anhydrous THF under an ice bath was slowly added NaH (102.4 mg, 4.26 mmol). The reaction mixture was stirred for about 2 hours, then quenched with water (70 ml) and neutralized with 2 M HCl. The mixture was diluted with EA (100 ml) and washed with water, saturated $NaHCO_3$ and brine. The organic phase was dried over anhydrous Na_2SO_4, concentrated and purified by flash chromatography (petroleum ether/ethyl acetate 50:1) to give 3D as light yellow oil (287 mg, 42%).^{1}H NMR (400 MHz, $CDCl_3$) δ 7.93 (d, *J* = 7.8 Hz, 2H), 7.57 (t, *J* = 7.4 Hz, 1H), 7.46 (t, *J* = 7.5 Hz, 2H), 4.19 (q, *J* = 7.1 Hz, 2H), 3.97 (s, 2H), 1.23 (t, *J* = 7.1 Hz, 3H).

(E, Z)-2-oxo-N’,2-diphenylacetohydrazonoyl cyanide(YuY)

To a stirred solution of aniline (93.13 mg, 1.11 mmol) in 2M HCl (2.5 mL, 5.0 mmol) under an ice bath was added $NaNO_2$ (99.56 mg, 1.45 mmol) in water (10 ml). 3-oxo-3-phenylpropanenitrile (3A) (161.8mg, 1.11mmol) in MeOH (10 ml) and NaOAc (833 mg, 10.16 mmol) in water (10ml) was mixed. The formed mixture was quickly added to the former solution within 5 minutes. The resulting solution was stirred at room temperature for about 1 hour. Yellow precipitate was formed. The reaction mixture was filtered, the filter cake was washed with PE and purified by column chromatography (petroleum ether/ethyl acetate 20:1) to give the product as a yellow solid (215mg, 78%). ^{1}H NMR (400 MHz, $CDCl_3$) δ 9.52 (s, 1H), 7.95 (d, *J* = 6.8 Hz, 2H), 7.55 (d, *J* = 5.5 Hz, 1H), 7.46 (d, *J* = 5.7 Hz, 2H), 7.34 (d, *J* = 6.3 Hz, 2H), 7.22 (d, *J* = 7.0 Hz, 2H), 7.15 (d, *J* = 6.5 Hz, 1H). MS (-ESI) m/z 248.0 $[M-H]^-$.

(E, Z)-2-(4-chlorophenyl)-2-oxo-N’-phenylacetohydrazonoyl cyanide (JP-1–31)

Compound *JP-1–31* was prepared from 3-(4-chlorophenyl)-3-oxopropanenitrile (3B) using the procedure described for 4A in 82% yield as a yellow solid.^{1}H NMR (400 MHz, $CDCl_3$) δ 9.70 (s, 1H), 7.94 (t, J = 6.8 Hz, 2H), 7.46 (s, 2H), 7.44 – 7.35 (m, 2H), 7.32 – 7.22 (m, 2H), 7.21 (t, *J* = 7.1 Hz, 1H). MS (-ESI) m/z 282.0 $[M-H]^-$.

(E, Z)-2-nitro-1-phenyl-2-(2-phenylhydrazono) ethanone (JP-1–32)

Compound *JP-1–32* was prepared from 2-nitro-1-phenylethanone (3C) using the procedure described for 4A in 60% yield as a yellow solid, which is a mixture of inseparable isomers (approximate ratio 2:1).For major one: ^{1}H NMR (400 MHz, $CDCl_3$) δ 12.09 (s, 1H), 7.95 (d, *J* = 8.2 Hz, 2H), 7.62 (s, 1H), 7.50 (t, *J* = 7.6 Hz, 2H), 7.44 (s, 2H), 7.35 (d, *J* = 7.5 Hz, 2H), 7.24 (s, 1H). For minor one: ^{1}H NMR (400 MHz, $CDCl_3$) δ 12.76 (s, 1H), 7.63 (s, 2H), 7.57 (t, *J* = 7.5 Hz, 1H), 7.40 (d, *J* = 11.9 Hz, 2H), 7.26 (s, 2H), 7.20 (d, *J* = 7.5 Hz, 2H), 7.17 (s, 1H). MS (-ESI) m/z 268.0 $[M-H]^-$.

(E, Z)-ethyl 3-oxo-3-phenyl-2-(2-phenylhydrazono)propanoate (JP-1–35)

Compound *JP-1–35* was prepared from ethyl 3-oxo-3-phenylpropanoate (3D) using the procedure described for 4A as a yellow oil, which is further crystallized with DCM and PE to give the product as a yellow solid in 47% yield.^{1}H NMR (400 MHz, $CDCl_3$) δ 12.75 (s, 1H), 7.93 (d, J = 7.8 Hz, 2H), 7.56 (t, J = 7.4 Hz, 1H), 7.47 – 7.41 (m, 2H), 7.29 (t, *J* = 7.6 Hz, 2H), 7.16 (d, *J* = 8.0 Hz, 2H), 7.07 (t, *J* = 7.2 Hz, 1H), 4.36 (q, *J* = 7.1 Hz, 2H), 1.34 (t, *J* = 7.1 Hz, 3H). MS (+ESI) m/z 319.0 $[M+Na]^+$.

SUPPLEMENTARY FIGURE

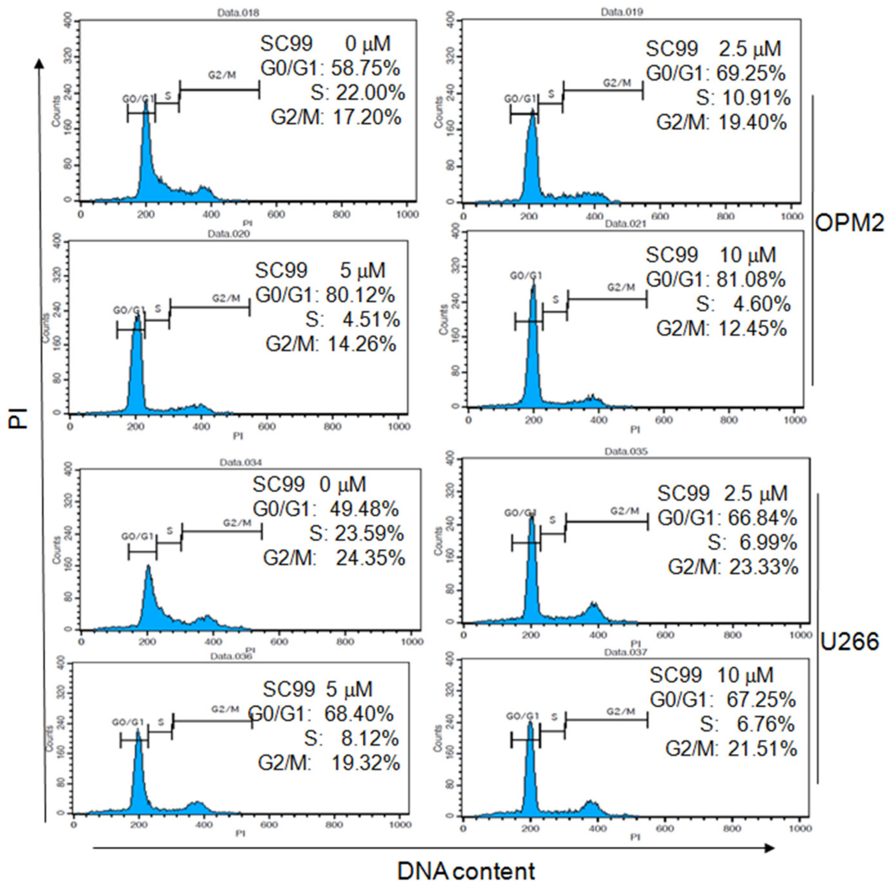

Supplementary Figure S1: SC99 arrests MM cells at the G0/G1 phase of cell cycle. Myeloma cells OPM2 and U266 were treated with increasing concentrations of SC99. Twenty-four hrs after incubation, cells were harvested, washed with cold phosphate buffered saline, suspended in 70% of cold ethanol, and incubated overnight at -20°C. Cells were washed and treated with 100 ng/mL DNase-free RNase at 37°C for 30 min. Cells were then stained with propidium iodide before being applied to cell cycle analysis on a flow cytometer.
